# Geometric and dosimetric effects of an automated clustering and plan optimization pipeline for multiple brain metastasis radiosurgery

**DOI:** 10.1002/acm2.70869

**Published:** 2026-10-20

**Authors:** Adam D. Yock, Guozhen Luo, Sean All, Austin N. Kirschner, Cornelis Kamerling

**Affiliations:** ^1^ Department of Radiation Oncology Vanderbilt University Medical Center Nashville Tennessee USA; ^2^ Brainlab SE Munich Bavaria Germany

**Keywords:** brain metastases, radiosurgery, SIMT, single‐isocenter multiple‐target, SRS

## Abstract

**Background:**

Treating multiple brain metastases simultaneously with single‐isocenter multi‐target (SIMT) stereotactic radiosurgery (SRS) can streamline treatment planning and delivery. However, it can also result in targets at large distances from the isocenter, increasing sensitivity to rotational setup errors and requiring wider collimator leaves. Grouping the targets and treating each group with its own plan retains the practical benefits of SIMT SRS while mitigating the concerns of targets at large distances off‐isocenter.

**Purpose:**

This work presents a prototype of a newly integrated treatment planning pipeline that automatically clusters targets into groups and subsequently optimizes a plan for each group simultaneously. Treatment scenarios featuring varying numbers of isocenters were compared.

**Methods:**

Twenty‐nine treatment courses for patients receiving SRS for 3–15 brain metastases were retrospectively analyzed. Multiple treatment scenarios were prepared for each patient‐course by grouping targets into 1–5 isocenters using k‐means clustering. PTV margins were added using two methods, one with a magnitude that varied with distance to the isocenter, and a second with a constant 1‐mm magnitude. A non‐clinical, command‐line version of the Brainlab Elements Multiple Brain Mets SRS software was used to optimize plans within each scenario simultaneously. Cumulative doses were analyzed in terms of geometric values (target distances to isocenter, total PTV volume), dosimetric values (volume of normal brain (brain‐GTVs) receiving 5, 8, 10, 12, and 18 Gy (V_5Gy_, V_8Gy_, V_10Gy_, V_12Gy_, and V_18Gy_)), and logistical values (number of Monitor Units (MUs)). A representative sample of 69% of plans with variable margins was reviewed by clinical SRS experts.

**Results:**

As the number of isocenters increased, distances from the targets to the isocenters decreased, and the proportion of targets within a given distance increased. For example, 36%, 77%, 94%, 99%, and 100% of targets were within 4 cm of the isocenter for 1–5 isocenters, respectively. For the variable margin method, the total PTV volume relative to the single‐isocenter scenario had a median decrease of 17.5, 24.3, 31.6, and 37.8% for 2–5 isocenters, respectively. Similarly, the relative change in the normal brain dose metrics decreased with increasing number of isocenters (e.g. V_12Gy_ decreased 20.1, 26.2, 36.2, and 40.9%, respectively). The relative change in dose metrics at each level was found to have a linear relationship with that of the total PTV volume (e.g. V_12Gy_, *R* ^2^ = 0.81). For the constant margin method, total PTV volume remained constant, but normal brain dose metrics still decreased due to the grouping and simultaneous plan optimization alone (e.g. V_12Gy_ decreased 6.4, 10.5, 15.1, and 17.1%). Regarding logistical values, the number of MUs increased with each additional isocenter for both margin methods, but at a rate slower than proportional to the number of isocenters. Of the evaluated plans, nearly all (97.7%) were clinically acceptable without modification.

**Conclusion:**

The SIMT SRS treatment planning pipeline presented here features automatic grouping of targets followed by automatic dose optimization of up to five isocenters. By quantifying the geometric, dosimetric, and logistical effects of adding isocenters, this pipeline can empower clinicians to make resource‐conscious treatment decisions optimal for each patient.

## 1 INTRODUCTION

Patients receiving stereotactic radiosurgery (SRS) for multiple brain metastases (mets) are increasingly treated with a single‐isocenter multi‐target (SIMT) technique that treats multiple radiosurgery targets with a common plan and isocenter.^1^, ^2^ Treating the targets simultaneously rather than sequentially with individual plans streamlines both the treatment planning and delivery process. The isocenters of plans treated with this technique are often placed in normal brain tissue among the set of targets to be treated, with the targets themselves being positioned at various distances off‐isocenter.

However, treating targets off‐isocenter introduces a number of disadvantages. The first is an increased sensitivity to rotational positioning errors. The accuracy of linear accelerators and image guidance systems, as well as patient setup tolerance limits, are typically characterized at the isocenter. However, errors of a given angular magnitude will correspond to increasingly large physical displacements as the distance from the isocenter increases, potentially compromising treatment delivery.^3^, ^4^, ^5^, ^6^ In addition, treating targets at large distances from the isocenter may require the use of wider multi‐leaf collimator (MLC) leaves that are less able to conform to small targets. Furthermore, at steep angles of beam divergence, the geometric and dosimetric characterization of the treatment beam and of MLC leaf end effects may be less precise. Lastly, smaller field sizes also reduce the area of the flattening filter, jaw edges, and MLC leaf ends that are exposed, decreasing the amount of scattered radiation reaching the patient.

Several common strategies are considered to address these issues, including: 1) limiting the maximum distance a target can be from the isocenter, 2) making patient setup tolerance limits more stringent for these cases, and/or 3) increasing target margins as a function of distance to the isocenter.^3^, ^4^, ^5^, ^6^, ^7^, ^8^, ^9^, ^10^, ^11^, ^12^, ^13^, ^14^, ^15^ To balance the advantages and disadvantages of the SIMT technique, clinicians will often look for natural geometric groupings of targets that might be well‐suited for a single plan with the targets remaining close to the isocenter. A patient's treatment may then consist of multiple plans—one for each grouping—but with the total number of plans still being considerably less than the total number of targets. The difficulty of grouping the targets can vary between patients, and is typically performed manually, which can be time intensive and can result in groupings that are user‐specific and that may be suboptimal. Methods to automatically group targets according to various metrics have been suggested but are not broadly commercially available or conveniently implemented into clinical systems and workflows.^5^ In addition, the dosimetric impact of these grouping methods on the resulting SRS plans has not been described in detail, leaving clinicians to guess at the true effects of these decisions on their treatment plans.

This work presents a prototype of an newly integrated SRS treatment planning pipeline that automatically groups brain mets to avoid large distances to the isocenter and then simultaneously optimizes an SRS plan for each group while considering the cumulative dose to the patient. If the desired number of SRS plans is known a priori, the pipeline can efficiently generate an optimal plan result. If the desired number of plans has not been determined, the pipeline can present alternatives with cumulative dose distributions composed of varying numbers of plans for evaluation. This work compares sets of SRS plans generated by the pipeline for an increasing number of target groups. Geometric and dosimetric characteristics of the plans are compared, along with their logistical implications regarding available clinical resources.

## 2 METHODS

### 2.1 Patient selection

For this retrospective study, patients that had previously received SRS for multiple brain mets at our clinic were considered through a reverse chronological review of our clinical database. It is common for these patients to undergo multiple courses of treatment that each treat multiple mets. The patient selection search was concluded once 40 patient‐courses were identified. This list was then filtered to include only patient‐courses that featured 3–15 mets treated in a single fraction. Clinical prescription doses ranged between 18–24 Gy. The result was 31 individual patient‐courses that represented the typical treatment scenarios observed in our clinic. Each of these patient‐courses was anonymized as an individual case using a clinical treatment planning system (Eclipse v16.1, Varian Medical Systems, Palo Alto, CA). Subsequently, the anonymization was manually reviewed for each case using an in‐house MATLAB script (vR2023a, Mathworks, Natick, MA) to ensure all identifying information had been removed. Each anonymized case was treated independently and is subsequently referred to as an individual “patient.”

### 2.2 Automatic clustering

The mets for each patient were automatically grouped using an in‐house SRS target clustering software program created using MATLAB (vR2023a). The program groups SRS targets using a k‐means clustering algorithm, which has been described for grouping brain mets previously.^5^ The user can select between an unweighted implementation of the algorithm or other implementations that are weighted in proportion to the target's volume, surface area, expected dosimetric coverage, amount of included normal tissue, or the inverse of these metrics. In this work, an unweighted implementation of the k‐means algorithm based on the geometric centroid of each met was used to cluster them into 1–5 groups, each to be treated with an individual SRS plan and isocenter. The maximum of 5 groups represents a practical limit that avoids an unnecessarily large number of plans to be generated and delivered while still maintaining relatively small distances between the targets and their isocenters.

In this work, the unweighted k‐means algorithm applied to the geometric centroids of the mets was repeated 100 times with various initialization states and allowed to run for a maximum of 100 iterations. The purpose of these repetitions was to generate a final result that was robust to the stochastic nature of the clustering algorithm. This large number of repetitions was likely not required for the system to converge to a consistent solution in cases with relatively few targets. However, they were used here nonetheless to promote robustness of the solution and because the time required to run the repetitions was effectively negligible.

### 2.3 Automatic planning

#### 2.3.1 Margins

After the mets were grouped for individual SRS plans and the isocenters were placed, isotropic planning target volume (PTV) margins were added to each target to account for positional uncertainty. Two different methods of determining the magnitude of those margins were explored in this work.

In the first method, the magnitude of the margin added to each met was determined as a function of the distance from its geometric centroid to the isocenter, as described in Equation 1 of Yock *et al*.^16^ Discrete values were calculated from this continuous function at 1‐cm increments from 0 to 9 cm. (Table 1). The minimum margin magnitude (1.00 mm) is consistent with margins commonly used for single‐isocenter single‐target treatments with no extra consideration for rotational uncertainty. The maximum margin magnitude was capped at the 9‐cm value (2.57 mm) because the treatment planning system used in this work can currently only accommodate ten individual values when automatically creating margins, and because margins this large warrant reconsidering the treatment planning technique. This work focused on margins adequate to address rotations of 1°, which corresponds to a common clinical action level for single‐target treatments. This was so that the experience of delivering the treatment would be more aligned with that of single‐target treatments and not require more stringent setup tolerances which tend to vary more between clinics and decrease the proportion of time the patient remains within tolerance. This method is most consistent with our current clinical practice in addressing rotational uncertainty in target positioning.

**TABLE 1:** Variable PTV margin expansions as a function of distance to isocenter.

Distance to Isocenter (cm)	Preferred Margin (mm)
0	1.00
1	1.17
2	1.35
3	1.52
4	1.70
5	1.87
6	2.05
7	2.22
8	2.40
≥ 9	2.57

The second method of adding PTV margins was to limit their magnitude to a constant 1‐mm expansion, regardless of target distance to the isocenter. While this method does not explicitly consider the increased rotational uncertainty known to geometrically affect targets that are off‐isocenter, it remains a common clinical practice even for SIMT SRS. In comparison with the first method, it also demonstrates the value of the automatic target grouping and simultaneous plan optimization pipeline even when maintaining a constant total PTV volume across different treatment scenarios.

#### 2.3.2 Optimization

Each treatment scenario featuring the 1–5 groups of mets and their isocenters was fed into an automatic treatment planning pipeline to optimize all plans simultaneously. All plans were generated using a non‐clinical, command‐line version of the Elements Multiple Brain Mets SRS software (v5.0 research version, Brainlab SE, Munich, Germany). For this work, this version of the software had been extended to allow for simultaneous multi‐isocenter optimization. First, parameters defining the treatment arc geometry were optimized for each group individually while avoiding the targets in other groups. This was achieved by a stochastic determination of the number of table angles, the positions of the table, the number of gantry arcs, and the span of the arcs based on the geometry of the beam's‐eye‐view and the PTVs. Then, the doses from a set of dynamic conformal arc (DCA) plans that each treated an individual group were all optimized simultaneously by incorporating the total summated dose into the objective function. The plans were normalized so that each target received at least 99.5% coverage to the PTV with a standardized prescription dose of 20 Gy. Optimization objectives and constraints were held constant across all isocenter scenarios and all patients to enable direct comparison of geometric and dosimetric effects. The plans were all created for a Varian Novalis TX linear accelerator featuring a high‐definition MLC and using a 6 MV x‐ray beam with a flattening filter. Dosimetric analysis was then performed on the summed dose from each group as described below, and the command‐line generated plans were imported into the Elements Multiple Brain Mets SRS software interface (v5.0 research version) for expert review.

### 2.4 Plan comparison and evaluation

The results of this automatic SRS treatment planning pipeline were compared both in terms of geometric and dosimetric plan characteristics. Geometrically, the different treatment scenarios featuring 1–5 isocenters were compared by calculating the proportion of mets that were within a given distance from the isocenter, and by measuring the total PTV volume normalized relative to the scenario where all mets were treated with a single isocenter. Dosimetrically, the volume of normal brain tissue receiving 5, 8, 10, 12, and 18 Gy (V_5Gy_, V_8Gy_, V_10Gy_, V_12Gy_, and V_18Gy_, respectively) were calculated. Normal brain tissue is defined here as the delineated brain structure minus each of the gross tumor volumes, and these dosimetric values were also normalized to the scenario where all mets were treated with a single isocenter. The decision to focus on the dose received by normal brain tissue and not discuss other common SRS plan metrics (e.g. conformity and gradient indices) was made to focus on the cumulative dose distribution of a treatment scenario and to avoid the complexities of reporting other metrics in the presence of bridging dose between targets.

As an indicator of the logistical considerations for delivering these plans, the number of Monitor Units (MUs) for each scenario was compared. The change in MUs is a reasonable proxy for the change in beam‐on delivery time if one assumes the treatment is delivered with a constant dose rate. It does not, however, consider other steps such as the repeated image‐guided patient alignment required for additional isocenters.

Finally, for each patient, the resulting dose from three of the five treatment scenarios optimized with the variable PTV margin method (87 summed dose distributions in total representing 69% of these plans) were evaluated by one of two radiation oncologists or a medical physicist that specialize in SRS. So as to review some instances of treatment scenarios with all possible numbers of isocenters, half of the patients were randomly selected to have the set of 1‐, 3‐, 5‐isocenter scenarios reviewed, while the other half had the set of 1‐, 2‐, 4‐isocenters scenarios reviewed. If the patient's small number of mets didn't allow for this review scheme (i.e. only had three or four mets), three available plans were still reviewed (e.g. 1‐, 2‐, 3‐isocenter scenarios). Dose distributions for plans optimized using the constant 1‐mm PTV margin were not reviewed by our clinical experts as this method was less consistent with our clinical process and represented total PTV volumes that were guaranteed to be smaller than those of the variable margin method. While review of a plan by only a single radiation oncologist or medical physicist does not constitute a comprehensive assessment, performing the review this way allowed us to increase the proportion of plans that received some kind of review by clinical experts and increased the likelihood of detecting undesirable plan characteristics. Clinical acceptability was judged based on institutional criteria consistent with conventional professional recommendations.

## 3 RESULTS

Of the filtered list of 31 patients intended to be used for this work, two had incomplete or erroneous configuration files that prevented adequate plan generation and evaluation by our pipeline, thus limiting the final data analysis to 29 patients. Among these patients, the volume of the mets before any margin had been added ranged from 0.01 to 1.36 cc with a mean of 0.17 cc (standard deviation = 0.24 cc). Figure 1 shows the distribution of the number of mets per patient. Thirteen of 29 patients (45%) had three or four mets each, while the remaining patients were distributed roughly uniformly in having 5–15 mets each. As we did not perform any explicit selection regarding the number of mets per patient beyond the inclusion range of 3–15, this suggests that the number of mets per patient typically observed in our clinic is skewed towards the low single digits.

**FIGURE 1:**
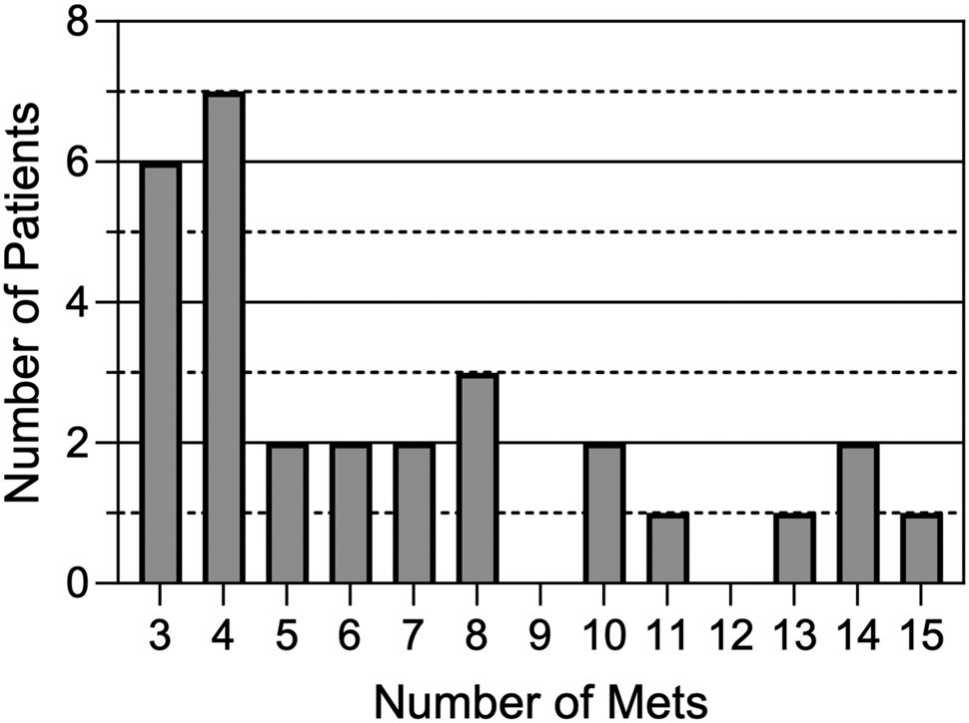
Distribution of the number of brain mets per patients.

Figure 2 presents the proportion of mets within given distances from their isocenter across scenarios that featured 1–5 plans and isocenters per patient. This figure illustrates that grouping the mets into an increasing number of plans increases the proportion of mets within a given distance of the isocenter. For example, roughly 36%, 77%, 94%, 99%, and 100% of mets were within 4 cm of their isocenter when treated with 1–5 plans, respectively. Similarly, it demonstrates that the median distance to the isocenter decreased from 4.7, 2.8, 2.0, 1.3, to 1.1 cm when treated with 1–5 plans. Also apparent in this figure is the diminishing effect of adding subsequent isocenters, with the change resulting from adding the second isocenter being greater than that of adding the fifth.

**FIGURE 2:**
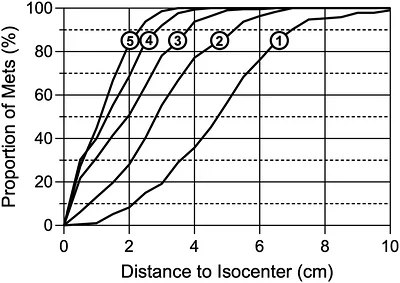
The proportion of mets within a given distance of their isocenter based on the number of plans and isocenters per patient (noted with the digits in the circular labels).

For plans generated using the variable PTV margin technique, a consequence of this systematic decrease in the distance between each met and its isocenter is the decrease in the total PTV volume as depicted in Figure 3. This is to be expected as the PTV margins were derived from a function that increases monotonically as a function of distance. Beyond this expected qualitative result, the magnitude of this effect is now quantified in Figure 3. Individual values were normalized to the treatment scenario featuring a single isocenter so that the change in the metric with additional isocenters could be clearly observed. Like the data in Figure 2, the change in the total PTV volume was observed to depict a diminishing effect with the increasing number of isocenters.

**FIGURE 3:**
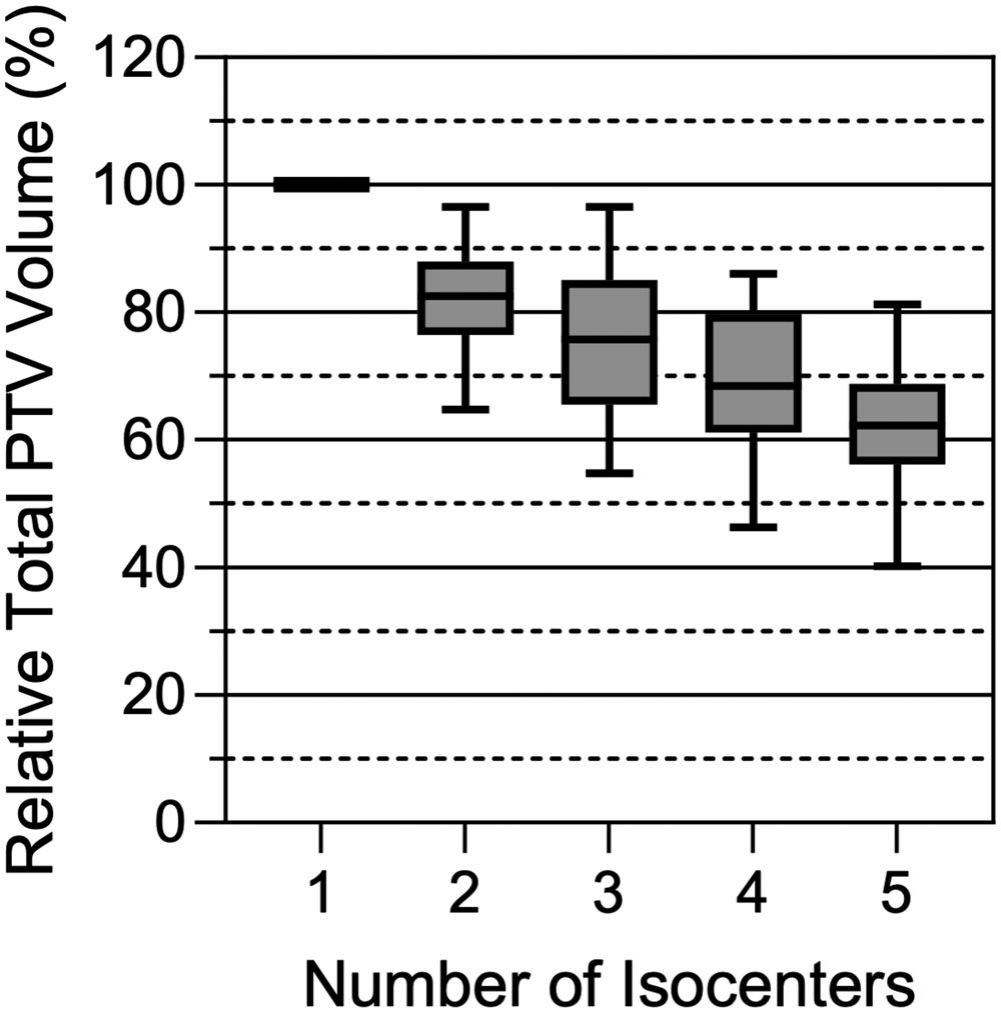
The relative change in total PTV volume as a function of the number of isocenters per patient for variable PTV margins. Values are normalized to the scenario with a single isocenter. The box represents the 25^th^‐75^th^ percentile inter‐quartile range while the whiskers extend from the minimum to the maximum value.

Successful dose optimization was, in part, verified by calculating the minimum coverage provided by the final plan. All targets were confirmed to be covered by the prescribed dose to at least 99.5% of the PTV. Figure 4 shows a change in the relative value of the V_12Gy_ to the normal brain (i.e. excluding gross tumor volumes, GTVs) for both PTV margin methods. As was the case in Figure 3, individual values were normalized to the treatment scenario featuring a single isocenter so that the change in the metric with additional isocenters could be clearly observed. The other dose metrics (V_5Gy_, V_8Gy_, V_10Gy_, and V_18Gy_) all behaved similarly and are presented in the Supplemental Materials. In the case of the variable PTV margin method, observing that a decrease in dose metrics followed the decrease in total PTV volume was also expected because the prescription dose of each plan was being fit to a shrinking target volume. However, Figure 4 illustrates that other potential effects of summing the dose from multiple plans together (e.g. the risk of increasing the integral dose or creating unintended bridging dose between targets) did not lessen the benefit of decreasing the total PTV volume. Even for the constant PTV margin method that featured no change in total PTV volume, the relative value of V_12Gy_ decreased (albeit with a smaller magnitude) just from the automatic grouping and simultaneous plan optimization. Performing a regression of the change in V_12Gy_ against that of the total PTV volume demonstrated that the dosimetric change was strongly correlated with the geometric change (*R* ^2^ = 0.81 for V_12Gy_, see Supplemental Materials for figures and data for all dose metrics).

**FIGURE 4:**
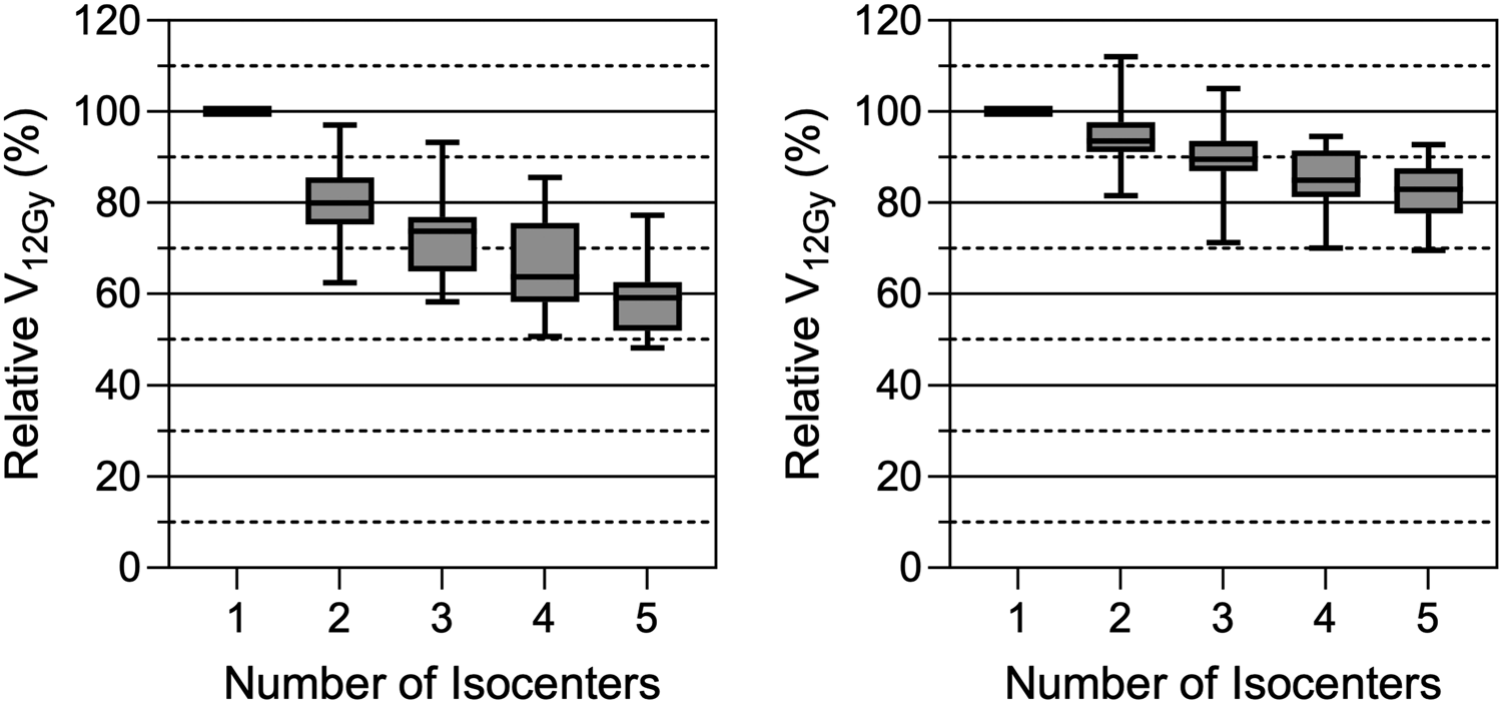
The relative change in V_12Gy_ as a function of the number of isocenters per patient. Values are normalized to the scenario with a single isocenter for variable PTV margins (left) and constant 1‐mm margins (right). See Supplemental Materials for similar analysis with V_5Gy_, V_8Gy_, V_10Gy_, and V_18Gy_. The box represents the 25^th^‐75^th^ percentile inter‐quartile range while the whiskers extend from the minimum to the maximum value.

Figure 5 presents the relative change in MUs as a function of the number of isocenters for both PTV margin methods. The number of MUs increased with each additional isocenter, but the increase was less than directly proportional to the number of isocenters. For example, the median change in MUs for a 3‐isocenter scenario compared to a single‐isocenter scenario was closer to 2x (rather than 3x), regardless of PTV margin method.

**FIGURE 5:**
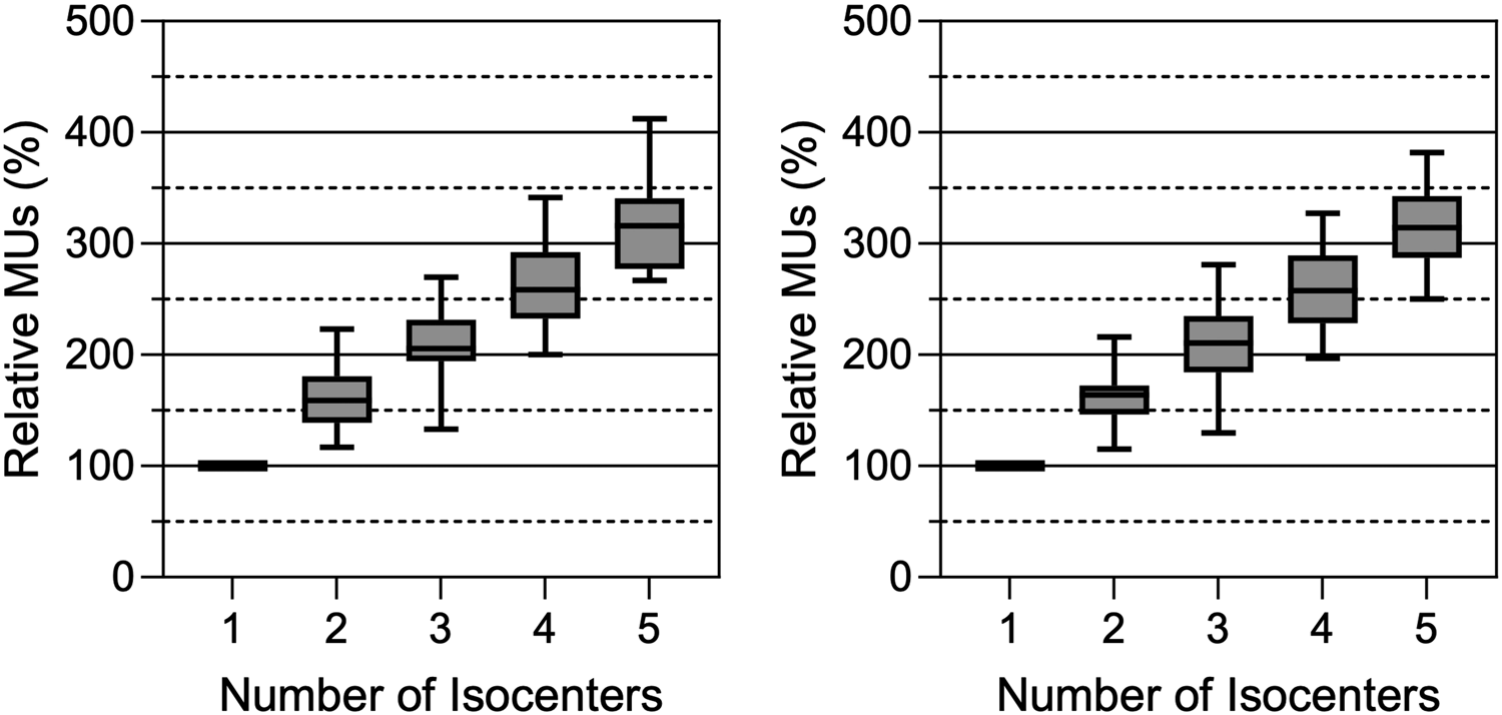
The relative change in MUs as a function of the number of isocenters per patient for variable PTV margins (left) and constant 1‐mm margins (right). Values are normalized to the scenario with a single isocenter. The box represents the 25^th^‐75^th^ percentile inter‐quartile range while the whiskers extend from the minimum to the maximum value.

Finally, for plans generated with variable PTV margins, clinical acceptability was assessed by expert clinician review using standard institutional criteria, including adequate target coverage, conformity, and normal tissue sparing. Plans were evaluated without modification. Of the 87 plans evaluated by clinical SRS specialists, which all used the variable PTV margin method, all but two of them (97.7%) were considered clinically acceptable without revision. For the remaining two of these plans (2.3%), the radiation oncologist reviewers preferred the plans be revised so as to further consider effects that were not explicitly prioritized by the general optimization objectives (e.g. further limiting dose to the scalp near shallow mets adjacent to the cranium).

## 4 DISCUSSION

In this work, we demonstrate that our SRS treatment planning pipeline that includes automatic target grouping with automatic simultaneous plan optimization can generate a set of high‐quality SRS plans featuring 1–5 treatment plans and isocenters with favorable geometric and dosimetric characteristics. Increasing the number of plans decreased the distance of mets to their respective isocenters, mitigating their sensitivity to rotational positioning errors. This, in turn, decreased the total PTV volume required to geometrically cover their variable position and subsequently decreased normal brain dose metrics, such as V_12Gy_. This not only decreases the risk of radionecrosis associated with an individual course of treatment, but is also pertinent as patients are increasingly being treated repeatedly with multiple courses of treatments, such that it can also decrease the overall longitudinal risk.

These gains can outweigh the incremental increase in the cost of clinical resources. However, the benefits of the treatment plans diminish with the addition of each successive isocenter, suggesting that at greater numbers of isocenters, practical costs (such as increased time on the treatment machine to setup, image, and verify each isocenter) may not be clinically justified. Incremental improvements in plan quality diminished with each additional isocenter, with the largest reductions in total PTV volume and normal brain dose metrics observed when changing from one to two isocenters. The incremental benefit from three to five isocenters was comparatively modest. These findings suggest the presence of a practical inflection point beyond which additional isocenters yield limited dosimetric benefit relative to their operational cost. This behavior was observed for both the variable PTV margin method and the constant 1‐mm PTV margin method, albeit the magnitude of the changes were less in the latter case because there was no change in the total PTV volume across the scenarios with different numbers of isocenters.

In addition, for plans generated with variable PTV margins, we demonstrate how the geometric and dosimetric effects of these plans were highly correlated, suggesting that the former could be considered a reliable proxy for the latter. This is advantageous because geometric considerations are generally available earlier on in the treatment planning process, prior to when plan optimization and dose considerations are determined. The use of an automated pipeline like that presented here can allow clinicians to determine the optimal balance between favorable plan characteristics and clinical resource utilization.

These findings offer a real‐life solution for choosing the isocenter number in patients suffering from multiple brain mets receiving SRS treatment. Reductions in target volumes and doses delivered to normal brain tissue can be explained by reduced distance between targets and their corresponding isocenters. This, in turn, decreased the required margin expansion. In addition, and even when constant PTV margins are used, optimizing the dose for all of the plans simultaneously leads to favorable cumulative dose distributions. From the clinical point of view, the greatest improvements in treatment plans can be achieved by adding a second or third isocenter. However, the number of isocenters should be selected individually based on patients’ characteristics, such as the number of lesions and their distribution, as well as the organizational aspects of treating patients at a particular institution. The pipeline described here facilitates appropriate clinical decision making specific to individual patients and the clinical environment they are treated in.

The choice made in this work to use an unweighted implementation of the k‐means clustering algorithm followed internal preliminary studies that compared the amount of normal tissue included within PTVs generated from different target groupings and isocenter positions. Some of the other algorithm implementations that were tested in‐house and featured weighted clustering schemes did further decrease the volume of normal tissue included in the PTVs compared to the unweighted implementation. However, it was observed that these improvements were small, and perhaps not justified when considering other geometric effects.

The findings presented in this work are consistent with related observations made in the literature. Several groups have described various methods to determine the position of the isocenter for SIMT SRS treatments. Methods include placing the isocenter in proportion to the targets’ centroid position,^4^, ^5^, ^15^, ^17^, ^18^, ^19^ volume,^4^, ^15^, ^16^ inverse volume,^4^, ^5^ surface area,^15^ or placing it through iterative numerical optimization of various metrics.^4^, ^13^, ^15^, ^16^, ^19^, ^20^ The effects of these methods are consistent throughout the literature in terms of their tendency to position the isocenter towards larger or smaller targets depending on the user's preference. Some groups prioritize maintaining target coverage in the presence of a given rotation, for example, by maximizing the overlap of the displaced target with its original position or the prescription isodose line. Because these metrics are normalized to the target volume, smaller targets are inherently more sensitive to rotations. These methods therefore tend to favor (i.e. move the isocenter in the direction of) smaller targets.^5^, ^13^, ^20^ Conversely, other groups prioritize minimizing the PTV, or relatedly, the volume of normal tissue included in the PTV required to cover the moving target. This objective leads to the opposite bias because more volume is added to the PTV when a given margin is added to a larger target compared to a smaller one. These methods tend to shift the isocenter towards the larger target.^15^, ^16^ Both of these methods move the isocenter away from the geometric center of the target centroids. While this may improve their particular objectives, it also increases the distance from the isocenter to the furthest target. This, in turn, incurs the other disadvantageous associated with the off‐isocenter targets mentioned in the Introduction. Positioning the isocenter at the geometric center of target centroids (as with the unweighted k‐means method) maintains short distances between each target and their isocenter, with particular emphasis on the distance to the furthest target.^4^, ^5^, ^19^

The primary use for the k‐means algorithm, however—even more than its use in finding an effective isocenter position for a group of targets—is grouping the targets to an associated isocenter in the first place. The most common automatic method described in the literature is an exhaustive search of all possible groupings. However, this method is more computationally intensive than the k‐means algorithm, particularly when considering the combinatorics of large numbers of targets, so it is only feasible for small numbers of targets. Kuo *et al.* describe a mixed‐method approach to the grouping task.^18^ When the number of targets and isocenters are both relatively low (below seven and three, respectively), an in‐house script automatically evaluated all possible clustering results to minimize the sum of squared Euclidean distance. For a greater number of targets, their method resorted to the k‐means algorithm to take advantage of its greater computational efficiency. Furthermore, when compared with an exhaustive search, the k‐means algorithm almost always produced the optimal grouping results. Hui *et al.* compared weighted and un‐weighted agglomerative clustering methods along with weighted and un‐weighted k‐means clustering methods, all relative to a ground truth result determined by an exhaustive search.^19^ They found that the un‐weighted k‐means result most closely matched the ground truth, differing from it in only one out of 126 scenarios (0.8%), and that was for a challenging case in which the patient had 29 targets. This demonstrates that the quality of grouping results from the k‐means algorithm in terms of minimizing the distance of targets to their isocenter and minimizing distance‐based‐margins does not need to be compromised for the algorithm's advantage of computational efficiency.

The magnitude of margins applied to SRS targets can vary considerably between specific clinical settings. While some clinicians may use 2 to 3‐mm margins on all mets,^21^ others argue that 0‐mm margins suffice.^22^ Ultimately, the appropriate margin depends heavily on the available technology and treatment procedures used (e.g. the magnitude of patient setup action levels or the use of intrafractional patient monitoring). The magnitudes of the variable margins presented in Table 1 are calculated based on the explicit geometry of an off‐isocenter target. These margins are what are mathematically required to completely cover targets that can move up to 1 mm and 1°. However, it may not be clinically required to cover such motion for every target all of the time. This suggests that smaller magnitude margins may suffice as long as the possibility of a geometric miss is accepted. In the work presented here, because we compared both a variable and a constant margin method, it may be possible to estimate the effects of intermediate margins by interpolating between our results. Furthermore, if complete target coverage is still preferred but the mathematically calculated margins seem excessive, then clinicians can use the techniques mentioned in the Introduction (e.g. more stringent patient setup tolerance limits) to decrease the sensitivity to rotational errors and thus the required margin. Our pipeline provides utility here as well by informing clinicians of the nonlinear effects of adding incremental isocenters to decrease the distance of targets to the isocenters and thus the distance‐based margins.

Several limitations should be considered when interpreting these results. This analysis focused on geometric and dosimetric endpoints without direct evaluation of clinical outcomes such as local control or radiation necrosis. The planning approach did not explicitly model rotational or translational setup errors beyond their incorporation into the distance‐based margin expansion. A further potential limitation of this work is the fairly simple geometric treatment of the mets during the grouping step. Grouping was achieved by considering the position of the mets but not their shape or volume. As described in the Methods section, this was the result of preliminary studies in which we decided that the benefits of intuitive isocenter placement and minimizing the maximum distance between each met and the isocenter outweighed the marginal decrease in total PTV volume achieved through weighted implementations of the k‐means clustering algorithm.

Another limitation is that the margin added to a target as a function of distance is applied isotropically. The reason for increasing the margin with distance is due to the increased sensitivity of off‐isocenter targets to rotational uncertainties. However, this increased uncertainty does not apply in the radial direction, only along the curved path traced out from rotations with their origin at the isocenter. The result is that the isotropic margin causes the PTV to be larger than it strictly needs to be in order to compensate for the true uncertainty. However, isotropic margins remain the default in the vast majority of clinical settings,^6^, ^7^, ^9^, ^10^, ^11^, ^12^, ^13^, ^14^ and curved margins designed to address this rotational uncertainty remain beyond the capabilities of most treatment planning systems.

An additional simplification made in this work was to ignore the proximity of mets to nearby organs‐at‐risk (OARs). Ultimately, the precise location of a met relative to an OAR is patient‐specific and needs to be considered on an individual basis. Our work, however, focused on conforming the dose tightly around the PTV, regardless of location, which proved to be an effective planning goal that generalized broadly across all patients. There are also clinical scenarios where not all brain mets may be desirable targets to include in the grouping step. If a target lies within the brainstem, it is often treated individually regardless of the presence of other mets, so as to minimize the extra dose to the surrounding tissue. Similarly, larger mets may warrant more treatment fractions than smaller ones, and combining them into a single grouping problem may not facilitate the dose‐fractionation schemes preferred for each target. Targets grouped together with a single isocenter can receive different doses, but the plan must be delivered in the same number of fractions. In these scenarios, clinicians can simply remove the target(s) in question from consideration in the grouping step, and reincorporate them during subsequent plan optimization and dose review.

We also did not include other common treatment planning metrics like the conformity or gradient indices. These are ultimately per‐target metrics. As such, there is additional complexity in determining how such metrics should best be treated when the local dose distribution may be influenced by the presence of another target nearby. Furthermore, the effect on any particular target of adding an isocenter may be to move the isocenter away from that target if doing so has a greater effect of reducing the distances to other targets in the group. Therefore, we expect the performance of individual target metrics to be more complex, ambiguous, and varied in their response than metrics like those for normal brain tissue dose, which are natively on the scale of the cumulative dose distribution.

Another important factor is how, during the automatic optimization of each set of plans, the contributions of all plans were considered simultaneously. By considering the dose from all plans, our pipeline avoided layering entrance and exit doses in unintended ways that can increase integral dose to the patient and dose bridging between targets. However, it is worth noting that, if not all plans are delivered in the same day, a particular target may become under‐covered if its full coverage depended on the contribution of dose from another plan that was ultimately delivered on a different day.

Lastly, this was a retrospective, single‐institution study. Our results are derived from the sample of patients treated at our own institution and selected for this study. We selected patients that were treated with 3–15 mets in a single course, with the majority having 5 or fewer mets. One should therefore exercise caution when extrapolating our results to numbers of targets beyond that reported here. However, our institutional experience has found that k‐means clustering is beneficial even for patients with several multiples of the number of targets considered in this work. More dramatic have been clinical cases whereby the number of mets have been so large that conventional SRS planning was prohibitive. Use of an automatic clustering tool, like that presented here, has effectively unlocked the ability to perform SRS when the patients would have otherwise received whole brain radiotherapy. As these patients do not fit the inclusion criteria of this study, their detailed description is beyond the scope of this work. While we feel the sample of patients studied here was largely representative of those at our institution with multiple brain mets that receive SRS with the SIMT technique, variations in treatment techniques between clinical institutions may affect the generalizability of our results.

Despite these limitations, our work demonstrates the value of automating target grouping and simultaneous plan optimization into an SRS treatment planning pipeline. With our pipeline, the process is guided by objective, quantitative metrics, not subjective heuristics that can depend on planner experience. If the desired number of target groups is known a priori, our pipeline can quickly generate a plan for each group with optimized geometric and dosimetric characteristics. If the desired number of target groups has not yet been determined, our pipeline provides a way to generate scenarios with various numbers of groups for comparison. Empowering clinicians with a streamlined way to generate one or more high‐quality SRS plans for patients with multiple brain mets is critical because the optimal clinical scenario is highly patient‐specific. Furthermore, these individual patient considerations must fit within the logistical constraints of the clinic. Clinics may or may not have the capacity to introduce additional isocenters for these patients, requiring additional time and staff involvement. The high acceptance rate by expert clinicians indicates the automatically generated plans met clinically relevant quality thresholds across a wide range of scenarios. While formal interobserver variability was not assessed, this result supports the feasibility of integrating automated planning pipelines into clinical workflows with minimal need for manual refinement. While we did not perform plan‐specific quality assurance to validate the deliverability of the numerous plans generated by our pipeline for this work, doing so for a plan selected to treat an actual patient would remain an important step in ensuring its quality and safety. Future work on the development of our pipeline is focused on exploring modulated treatment deliveries, evaluating additional planning metrics, and directly quantifying the time and cost savings of our pipeline.

This work provides a practical framework for decision‐making in the treatment of multiple brain mets by quantifying the relationship between isocenter number, geometric configuration, and dosimetric outcomes. Clinicians can use these results to anticipate the magnitude of improvement achievable with additional isocenters and to weigh these gains against the associated increase in treatment complexity, rather than relying solely on planner experience or heuristic approaches. This ability to systematically generate and evaluate multiple planning scenarios is particularly valuable in the context of increasing patient volumes and resource constraints, where efficient yet high‐quality treatment delivery remains a central priority.

Even without direct access to this pipeline, clinicians can benefit from the results presented in this work because of the new quantitative and dosimetric data it provides along with the associated patterns and correlations. Regardless of the grouping method used or planning system available, a clinic can compare the distances to the targets they wish to treat with our observations about how these change with additional isocenters. The clinic could then determine what this implies for changes in the total PTV volume, and due to the high correlation between geometric and dosimetric values demonstrated here, estimate certain treatment plan properties.

## 5 CONCLUSION

This study demonstrates that a prototype of a newly integrated automated SRS treatment planning pipeline that incorporates target grouping and simultaneous optimization can efficiently generate sets of high‐quality SRS plans for patients with multiple brain mets treated with the SIMT technique across a range of isocenter configurations. These results quantify the effect that adding successive isocenters has on decreasing the distance of targets to their isocenter, on decreasing the total PTV volume, and on decreasing several important dose metrics such as normal brain V_12Gy_. The results depict the magnitude of these changes, the tendency for the effect size to diminish when adding successive isocenters, and the strong correlation between the geometric and dosimetric characteristics of the plans. These findings further highlight the importance of balancing dosimetric advantages with the logistical and operational costs associated with additional isocenters. By explicitly quantifying tradeoffs, this work provides clinically relevant data and a framework to guide isocenter selection and supports more informed, patient‐specific treatment planning decisions in the management of multiple brain mets.

## AUTHOR CONTRIBUTIONS

ADY contributed to the conceptualization, planning, data acquisition, and data analysis for this project, as well as the drafting and editing of the manuscript. GL contributed to the conceptualization, planning, and data acquisition for this project, as well as the editing of the manuscript. SA contributed to the data acquisition for this project, as well as the editing of the manuscript. ANK contributed to the data acquisition for this project, as well as the editing of the manuscript. CK contributed to the conceptualization, planning, data acquisition, and data analysis for this project, as well as the drafting and editing of the manuscript.

## CONFLICT OF INTEREST STATEMENT

ADY reports copyrighted software, a patent‐pending algorithm, and speaker travel support and honorarium pertaining to the work presented in this manuscript. ADY also reports patents, both granted and pending, regarding an adaptive radiotherapy phantom unrelated to this work.

## Supporting information




**Supporting Information**: acm270869‐sup‐0001‐FiguresS1‐S9.docx

## Data Availability

Authors are not able to share data at this time.
