# Supplementary material for: Geometric and dosimetric effects of an automated clustering and plan optimization pipeline for multiple brain metastasis radiosurgery
Source: J Appl Clin Med Phys. 2026 Oct 20;27(11):e70869. doi: 10.1002/acm2.70869 (PMC13682620; doi:10.1002/acm2.70869)
Supplement: Supplementary file 1 [file ACM2-27-e70869-s001.docx]

Supplemental Materials

Supplemental Figure 1. The relative change in V_5Gy_ as a function of the number of isocenters per patient for variable PTV margins (left) and constant 1-mm margins (right). Values are normalized to the scenario with a single isocenter. The box represents the 25^th^-75^th^ percentile inter-quartile range while the whiskers extend from the minimum to the maximum value.

Supplemental Figure 2. The relative change in V_8Gy_ as a function of the number of isocenters per patient for variable PTV margins (left) and constant 1-mm margins (right). Values are normalized to the scenario with a single isocenter. The box represents the 25^th^-75^th^ percentile inter-quartile range while the whiskers extend from the minimum to the maximum value.

Supplemental Figure 3. The relative change in V_10Gy_ as a function of the number of isocenters per patient for variable PTV margins (left) and constant 1-mm margins (right). Values are normalized to the scenario with a single isocenter. The box represents the 25^th^-75^th^ percentile inter-quartile range while the whiskers extend from the minimum to the maximum value.

Supplemental Figure 4. The relative change in V_18Gy_ as a function of the number of isocenters per patient for variable PTV margins (left) and constant 1-mm margins (right). Values are normalized to the scenario with a single isocenter. The box represents the 25^th^-75^th^ percentile inter-quartile range while the whiskers extend from the minimum to the maximum value.

Supplemental Figure 5. Correlation between the relative change of V_5Gy_ and the relative change of the total PTV volume for variable PTV margins.

Supplemental Figure 6. Correlation between the relative change of V_8Gy_ and the relative change of the total PTV volume for variable PTV margins.

Supplemental Figure 7. Correlation between the relative change of V_10Gy_ and the relative change of the total PTV volume for variable PTV margins.

Supplemental Figure 8. Correlation between the relative change of V_12Gy_ and the relative change of the total PTV volume for variable PTV margins.

Supplemental Figure 9. Correlation between the relative change of V_18Gy_ and the relative change of the total PTV volume for variable PTV margins.
